# Short-term glucocorticoid excess blunts abaloparatide-induced increase in femoral bone mass and strength in mice

**DOI:** 10.1038/s41598-021-91729-8

**Published:** 2021-06-10

**Authors:** Mikkel Bo Brent, Jesper Skovhus Thomsen, Annemarie Brüel

**Affiliations:** Department of Biomedicine, Health, Aarhus University, Wilhelm Meyers Allé 3, 8000 Aarhus C, Denmark

**Keywords:** Osteoporosis, Diseases, Endocrine system and metabolic diseases

## Abstract

Glucocorticoids (GCs), such as prednisolone, are widely used to treat inflammatory diseases. Continuously long-term or high dose treatment with GCs is one of the most common causes of secondary osteoporosis and is associated with sarcopenia and increased risk of debilitating osteoporotic fragility fractures. Abaloparatide (ABL) is a potent parathyroid hormone-related peptide analog, which can increase bone mineral density (aBMD), improve trabecular microarchitecture, and increase bone strength. The present study aimed to investigate whether GC excess blunts the osteoanabolic effect of ABL. Sixty 12–13-week-old female RjOrl:SWISS mice were allocated to the following groups: Baseline, Control, ABL, GC, and GC + ABL. ABL was administered as subcutaneous injections (100 μg/kg), while GC was delivered by subcutaneous implantation of a 60-days slow-release prednisolone-pellet (10 mg). The study lasted four weeks. GC induced a substantial reduction in muscle mass, trabecular mineral apposition rate (MAR) and bone formation rate (BFR/BS), and endocortical MAR compared with Control, but did not alter the trabecular microarchitecture or bone strength. In mice not receiving GC, ABL increased aBMD, bone mineral content (BMC), cortical and trabecular microarchitecture, mineralizing surface (MS/BS), MAR, BFR/BS, and bone strength compared with Control. However, when administered concomitantly with GC, the osteoanabolic effect of ABL on BMC, cortical morphology, and cortical bone strength was blunted. In conclusion, at cortical bone sites, the osteoanabolic effect of ABL is generally blunted by short-term GC excess.

## Introduction

Glucocorticoids are widely used to treat inflammatory conditions and diseases^1,2^. Oral glucocorticoids, such as prednisolone, are thus a cornerstone in all phases of immunosuppression and are extensively used to treat rheumatic^3^, dermatological^4^, vascular^5^, renal^6^, and pulmonary diseases^7^ as well as cancer^8^. However, continuous long-term treatment with glucocorticoids induces a series of adverse effects including severe influences on the musculoskeletal system like rapidly decreased muscle mass and bone density, which subsequently increases the risk of debilitating osteoporotic fragility fractures^9,10^.

Glucocorticoid-induced osteoporosis is one of the most common causes of secondary osteoporosis^10^. It is estimated that 1–3% of adults in the United States use glucocorticoids at any given time with a mean treatment duration of more than 4 years^11^. Although oral glucocorticoids are widely used in the United States, anti-osteoporotic pharmaceutical treatment is only reported by less than 40% of oral glucocorticoid users, which underlines the existence of barriers for sufficient osteoporosis management in these patients^12,13^.

Several pharmaceutical countermeasures for glucocorticoid-induced osteoporosis have been investigated in large multicenter studies of alendronate^14,15^, zoledronic acid^16^, teriparatide (PTH [1–34])^17^, or denosumab and risedronate^18^. Patients with severe glucocorticoid-induced osteoporosis (T-score ≤  − 3.5 or T-score ≤  − 2.5 plus a fragility fracture) are usually candidates for treatment with teriparatide, since daily injections of 20 µg teriparatide results in a greater increase in bone mineral density (BMD) than treatment with alendronate^17^. However, treatment with teriparatide has been associated with an increased risk of hypercalcemia, since it not only stimulates bone formation, but also bone resorption^19^. In addition, studies of rodents have suggested the osteoanabolic effect of teriparatide is blunted by concomitant treatment with glucocorticoids^20,21^, therefore, alternative bone anabolic treatment options are needed.

The parathyroid hormone-related protein analog abaloparatide (ABL) is a bone anabolic agent with properties similar to teriparatide, but entails a lower risk of inducing hypercalcemia in patients^22^. Consequently, ABL might be an alternative treatment option for selected patients at risk of hypercalcemia. ABL interact with the parathyroid hormone 1 receptor (PTH1R) expressed on osteoblasts, osteocytes, bone lining cells, and other mesenchymal-derived cells^23,24^. Intermittent interaction with PTH1R initiates an intracellular signaling cascade that ultimately result in bone formation, particularly at trabecular bone sites, and to a lesser extend osteoclastic bone resorption^25–27^. Since ABL and teriparatide are structurally similar and acts through the same receptor, blunting of the osteoanabolic effect may also be a concern for ABL.

The study aimed to investigate whether GC excess blunts the osteoanabolic effect of ABL in mice.

## Material and methods

### Animals and treatments

Sixty female outbred RjOrl:SWISS mice were purchased from Janvier Labs (Le Genest-Saint-Isle, France) and allowed two weeks of acclimatization before the study started. At study start, the mice were 12–13 weeks old, had a mean bodyweight (BW) of 31.8 ± 2.3 g, were housed in standard plastic mice cages with 5–6 animals per cage, and maintained at 21 °C with a 12:12 h light/dark cycle (6:00 am lights on/ 6:00 pm lights off). During the study, all mice had unrestricted access to tap water and standard mice chow (1324 maintenance diet for rats and mice; Altromin, Lage, Germany). Five days before the study start, the mice were stratified using custom-made software into the following groups based on their BW: Baseline, Placebo-pellet + saline (Control), Placebo-pellet + abaloparatide (ABL), glucocorticoid-pellet + saline (GC), and glucocorticoid-pellet + abaloparatide (GC + ABL). Animals assigned to the Baseline group were sacrificed at the study start to enable determination of age-dependent musculoskeletal changes.

60-days slow-release glucocorticoid-pellets (prednisolone) and placebo-pellets were acquired from Innovative Research of America (Sarasota, FL, USA) and inserted subcutaneously. The glucocorticoid-pellets were designed to release 10 mg of prednisolone over a period of 60 days (equivalent to 5.4 mg/kg/d) by erosion and diffusion, and established a local concentration gradient. Placebo-pellets contained vehicle and no active agent. Under general anesthesia by 3% inhaled isoflurane, a 0.5-cm-long incision was made at the midline between the shoulder blades using a surgical-grade lancet and the pellet was placed at the lateral side of the neck. The incision was closed using resorbable multifil suture (Eickemeyer, Tuttlingen, Germany) and LiquiBand skin adhesive (Plymouth, Devon, UK) if needed.

ABL (H-8334, Bachem, Bubendorf, Switzerland) was dissolved in 0.9% isotonic saline and administered (100 μg/kg/day) as subcutaneous injections five days a week to animals in the ABL and GC + ABL groups for four weeks. Likewise, mice allocated to the Control and GC groups were injected subcutaneously five days a week with a similar amount of saline.

To assess bone resorption and formation, all mice were injected subcutaneously with tetracycline (20 mg/kg, T3383, Sigma-Aldrich, St. Louis, MO, USA) four days before study start and with calcein (20 mg/kg, C0875, Sigma-Aldrich, St. Louis, MO, USA) and alizarin (20 mg/kg, A3882, Sigma-Aldrich, St. Louis, MO, USA) four and eight days before sacrifice, respectively. The tetracycline served as a baseline label enabling estimation of bone erosion throughout the experiment as previously described^28,29^, while the calcein and alizarin labels allowed for the determination of conventional bone formative parameters at the end of the trial.

After four weeks of treatment, the mice were sacrificed by exsanguination under general anesthesia with isoflurane. One mouse in the GC group was sacrificed before the study end due to infection at the incision site and failure to thrive.

The study was carried out in compliance with the ARRIVE guidelines^30^ and all methods were performed in accordance with relevant guidelines. All experimental protocols for the study were approved by the Danish Animal Experiment Inspectorate (2018-15-0201-01436).

### Tissue extraction and femoral length

Immediately after exsanguination, both femora, the right tibia, and L4 were isolated, and soft tissue was carefully removed. The right femur, right tibia, and L4 were stored in Ringer’s solution at − 20 °C, while the left femur was immersion-fixed in 0.1 M sodium phosphate-buffered formaldehyde (4% formaldehyde, pH 7.0) for 48 h and then stored in 70% ethanol.

The femoral bone length was determined using a digital sliding caliper and measured from the medial condyle to the top of the femoral head.

### Rectus femoris muscle

The right rectus femoris muscle was removed in a standardized manner, and the wet weight was determined immediately using a digital scale (Mettler AT250, Columbus, OH, USA). Then, the muscles were halved at the midpoint, placed with the cut surface on a flat-bed image scanner (Perfection 3200 Photo; Seiko Epson, Nagano, Japan), and scanned to determine the whole muscle cross-sectional area (CSA)^31^.

The halved rectus femoris muscle was then immersion-fixed in 0.1 M sodium phosphate-buffered formaldehyde (4% formaldehyde, pH 7.0) and embedded in plastic-based 2-hydroxyethyl methacrylate (Technovit 7100, Heraeus Kulzer, Wehrheim, Germany). The embedded muscles were cut into 2-μm-thick sections on a microtome (Jung RM2065; Leica Instruments, Nussloch, Germany) and stained with Masson’s trichrome to determine muscle cell CSA. Muscle cell CSA was determined using a light microscope (Nikon Eclipse i80, Tokyo, Japan) at a final magnification of × 1132 as previously described^29^.

### Dual-energy X-ray absorptiometry (DXA)

Areal bone mineral density (aBMD) and bone mineral content (BMC) were determined using a desktop DXA (pDEXA Sabre XL; Norland Stratec, Pforzheim, Germany) as previously described^32^. In brief, the whole femur and tibia were scanned using a pixel size of 0.1 × 0.1 mm^2^ and a scan speed of 3.0 mm/s.

### Micro computed tomography (μCT)

**Figure 1:**
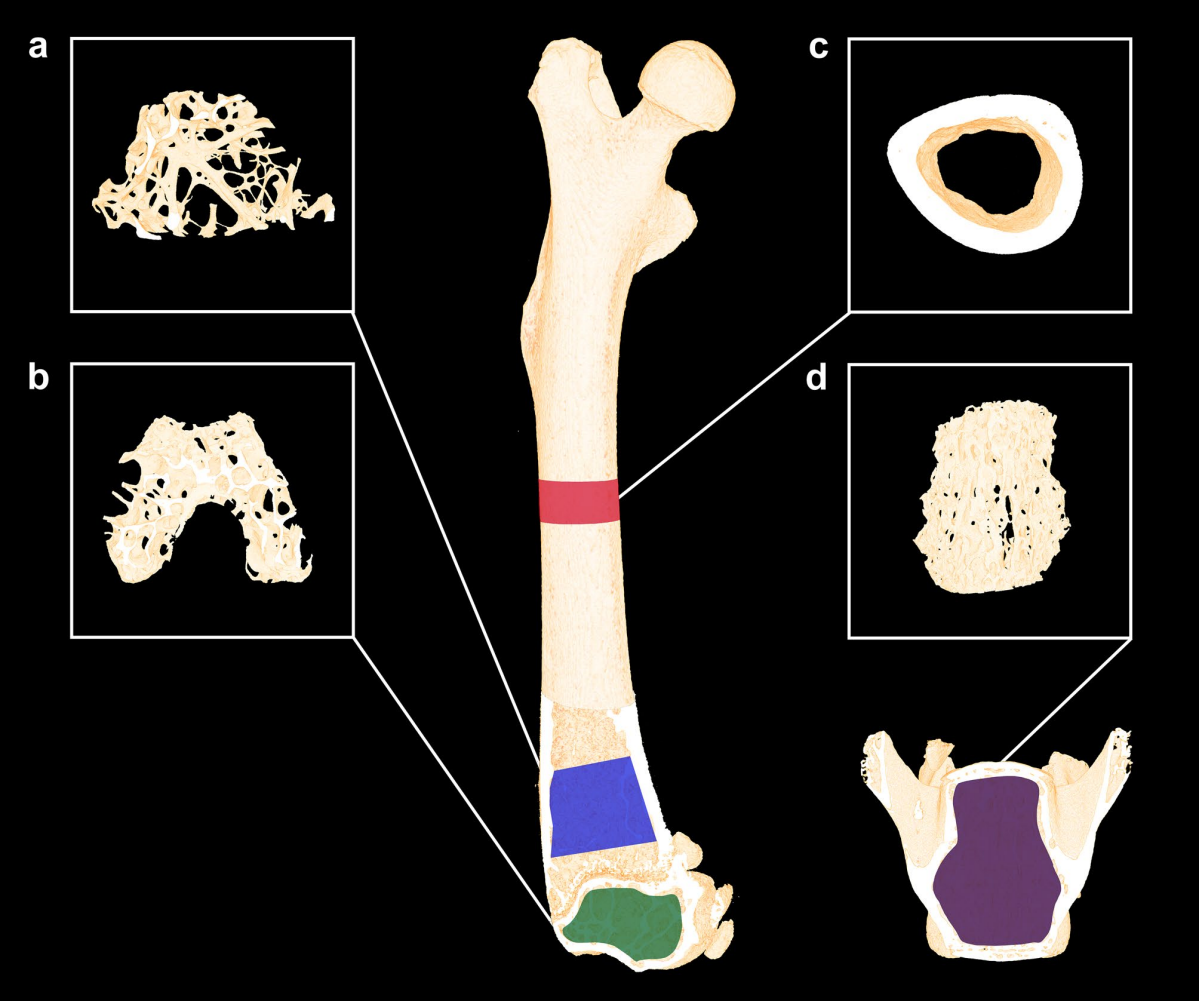
Skeletal sites investigated with μCT using a voxel size of 3.5 μm. (**a**) Blue represents the 1,000-μm-high volume of interest (VOI) at the distal femoral metaphysis. (**b**) Green represents the VOI at the distal femoral epiphysis (approximately 490-μm-high). (**c**) Red represents the 820-μm-high VOI at the femoral mid-diaphysis. (**d**) Purple represents the VOI at the L4 vertebral body (approximately 2000-μm-high). Dimensions are not to scale.

Cortical and trabecular microarchitectural bone parameters of the distal femoral metaphysis and epiphysis and the vertebral body of L4 were determined using a desktop µCT scanner (Scanco µCT 35; Scanco Medical, Brüttiselen, Switzerland) (Fig. 1). All scans were performed in high-resolution mode (1000 projections/180°) with an isotropic voxel size of 3.5 μm, an X-ray tube voltage of 55 kV, a current of 114 μA, and an integration time of 800 ms. Beam hardening artifacts were reduced using a 0.5 mm aluminum filter.

The bone samples were analyzed using separate volumes of interest (VOIs) as previously described^27^ (Fig. 1). The VOI at the femoral mid-diaphysis comprised an 820-μm-high region (234 slices) centered on the femoral mid-point, thus containing cortical bone only. The VOI at the distal femoral metaphysis started 200 μm (57 slices) above the point, where the mineralized cartilage from the growth plate fused and ended 1000 μm (229 slices) further above and contained trabecular bone only. The femoral epiphysis was analyzed using a 490-μm-high (140 slices) VOI that started just after the medial and lateral epicondyle have fused to a coherent structure and ended, where the growth plate first appeared, thus containing trabecular bone only. Finally, L4 was analyzed using an approximately 2000-μm-high (571 slices) VOI spanning from the upper to the lower growth plate excluding primary spongiosa containing trabecular bone only. The data were low-pass filtered using a Gaussian filter (σ = 0.8 and support = 1) and segmented with a fixed threshold filter determined with IPL (v. 5.11, Scanco Medical AG, Switzerland). The thresholds used for segmentation were 574 mg HA/cm^3^ for femoral bone and 529 mg HA/cm^3^ for L4.

The assessment of bone microstructure followed the current guiding principles by Bouxsein et al.^33^. The following microstructural bone parameters were assessed with µCT: Ct.Th: cortical thickness, B.Ar: bone area, M.Ar: marrow area, T: tissue area, Ct.Po: cortical porosity, BV/TV: bone volume/tissue volume, Tb.Th: trabecular thickness, Tb.Sp: trabecular spacing, Tb.N: trabecular number, CD: connectivity density, SMI: structure model index, vBMD: volumetric bone mineral density, and TMD: tissue mineral density.

### Mechanical testing

Mechanical properties of the femoral mid-diaphysis, femoral neck, and vertebral body of L4 were determined using a material testing machine (Instron model 5566, United Kingdom) as previously described^31,32^. Maximum load to failure was the ultimate load achieved at any point during the compression testing and was determined from the load–displacement data using in-house developed software. The femur was placed on two rounded supporting bars separated by 7.14 mm with the anterior side facing away from the supporting bars for the 3-point bending test. Load was applied with a third rounded bars at the femoral mid-point. Then, the proximal part of the femur was placed in a custom-made fixation device exposing the femoral neck for mechanical testing. L4 was compression tested after the vertebral disc and processi were removed. For all testing procedures, vertical load was applied at a constant deflection rate of 2 mm/min.

### Dynamic bone histomorphometry

#### Cortical bone

After mechanical testing, a 200-μm-thick mid-diaphyseal cross-sectional bone slice was sawed from the distal part of the femoral bone and mounted with Pertex on glass slides. Fluorochrome bone labels were analyzed using a brightfield microscope equipped for fluorescence (Nikon Eclipse i80, Tokyo, Japan). Live images were projected to a computer equipped with the stereological software Visiopharm (v. 2020.09, Visiopharm, Hørsholm, Denmark). A 24-arm circular grid radiating outwards from the center of the bone marrow was superimposed on live images to quantify fluorochrome labels. The distance between double labels was measured using a built-in measure tool in the Visiopharm Software. Mineralizing surface/bone surface (MS/BS), mineral apposition rate (MAR), and bone formation rate (BFR/BS) was determined as previously described^29^. In the case of missing double labels an imputed value of zero was used to calculate MAR^34^. Tetracycline-covered surfaces (Tetra.S/BS) were used as an indicator of bone resorption throughout the experiment^28,29^. The assessment of fluorescent labels was performed at a final magnification of × 1132.

#### Trabecular bone

**Figure 2:**
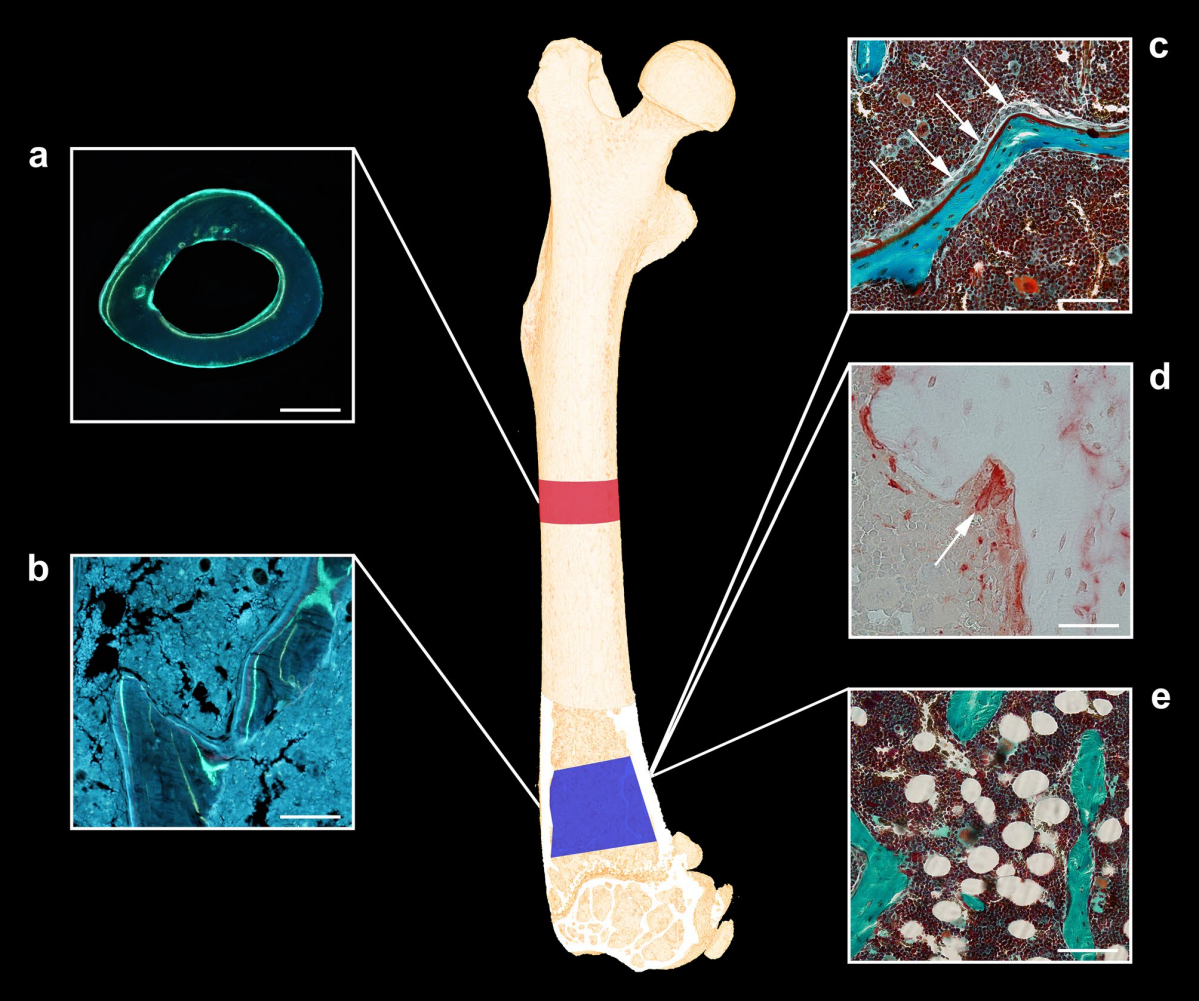
(**a**) Dynamic bone histomorphometry at the femoral mid-diaphysis. Bar = 0.5 mm. (**b**) Dynamic bone histomorphometry at the distal femoral metaphysis. Bar = 50 μm. (**c**) Osteoblasts (white arrows) residing on red osteoid at the distal femoral metaphysis. Masson–Goldner trichrome. Bar = 50 μm. (**d**) TRAP-stained osteoclast in a Howship’s lacuna at the distal femoral metaphysis. Bar = 100 μm. (**e**) Unilocular adipocytes in the bone marrow at the distal femoral metaphysis. Masson–Goldner trichrome. Bar = 50 μm.

The distal part of the femur was embedded undecalcified in methacrylate and 7-μm-thick longitudinal sections were cut using a microtome (Jung RM2065; Leica Instruments, Nussloch, Germany). The sections were either left unstained for dynamic bone histomorphometry, Masson–Goldner trichome-stained for osteoblast, osteoid, and adipocyte quantification or stained for tartrate-resistant acid phosphatase (TRAP) to identify osteoclasts (Fig. 2). Fluorochrome labels were quantified in a 1000-μm-high region of interest (ROI) starting 300 μm below the most distal part of the growth plate. The fields of view were sampled systematically covering 100% of the ROI with a randomly orientated line grid superimposed on the fields of view. The assessment of fluorescent labels was performed at a final magnification of × 1132.

### Bone cells and adipocytes

The Masson–Goldner trichome-stained sections from the distal part of the femur were used to estimate the amount of osteoblast (Ob.S/BS), osteoid-covered surfaces (OB/BS), bone marrow adiposity, and adipocyte mean size. Osteoblasts were defined as cuboidal cells residing at the bone surface and osteoid was defined as the red unmineralized region running along the edge of the bone.

Using a point grid, the bone marrow adiposity (adipocyt volume density) was estimated as the total number of points hitting adipocytes within the bone marrow divided by total number of point hitting the bone marrow. Adipocytes were defined as cells containing a single large lipid droplet surrounded by a layer of cytoplasm^35^.

Sections stained for TRAP were used to determine the amount of osteoclast-covered surfaces (Oc.S/BS). Osteoclasts were defined as TRAP-positive cells located at the bone surface.

A similar ROI, final magnification, and superimposed randomly orientated line grid used for trabecular dynamic bone histomorphometry were used to analyze the sections stained with Masson–Goldner trichome and the sections stained for TRAP.

### Statistics

Statistical analysis was conducted in GraphPad Prism 9.2 (Systat Software, Chicago, IL, USA). Differences between groups were analyzed using a parametric two-way analysis of variance (ANOVA) followed by a post-hoc Holm-Sidak test. Normality was assessed using the D'Agostino-Pearson test. The mean of all groups was compared to the mean of every other group, except Baseline. The two-way ANOVA is capable of detecting interactions, wherein a significant interaction indicates a difference in the effect of treatment with ABL between mice with a placebo-pellet (Control) or a glucocorticoid-pellet (GC) implanted. Data are presented as means ± SD and statistical significance was considered achieved if the *p*-value was less than 0.05. An a priori sample size calculation (power = 0.8) on mice showed that a 5% difference in femoral aBMD and a 15% difference in mid-diaphyseal bone strength can be demonstrated between groups with *n* = 12 animals^27^.

## Results

### Animals, muscle mass, and bone length

The final body weight was not affected by injections with ABL or insertion of subcutaneous GC-pellets compared with Control (Table 1).

**Table 1:** Number of animals, initial and final bodyweight (BW), rectus femoris cross-sectional area (CSA), femoral bone length, aBMD (areal bone mineral density), and BMC (bone mineral content) of mice subjected to subcutaneous slow-release glucocorticoid (GC) pellets and treated with abaloparatide (ABL) for four weeks. Mean ± SD. *p < 0.05 vs. Control. #*p* < 0.05 vs. ABL.

	Baseline	Control	ABL	GC	GC + ABL	Interaction
Animals	12	12	12	11	12	No
Initial BW (g)	31.5 ± 2.27	31.3 ± 2.30	31.9 ± 2.20	32.5 ± 2.97	31.7 ± 2.07	No
Final BW (g)	31.5 ± 2.3	30.9 ± 2.21	32.9 ± 2.18	30.7 ± 2.83	29.5 ± 2.91^#^	No
Rectus femoris muscle CSA (cm^2^)	1.46 ± 0.18	1.40 ± 0.14	1.39 ± 0.14	1.18 ± 0.26*^,#^	1.05 ± 0.23*^,#^	No
Femoral bone length (mm)	15.7 ± 0.44	15.8 ± 0.49	16.1 ± 0.51	16.0 ± 0.26	15.8 ± 0.57	No
Femoral aBMD (mg/cm^2^)	79.3 ± 7.75	79.4 ± 5.91	88.9 ± 9.46*	73.1 ± 6.53^#^	77.6 ± 6.30^#^	No
Femoral BMC (mg)	29.9 ± 3.12	30.4 ± 2.74	36.9 ± 4.00*	28.8 ± 3.38^#^	31.0 ± 2.03^#^	Yes *p* = 0.023
Tibial aBMD (mg/cm^2^)	65.9 ± 5.10	69.9 ± 5.59	79.1 ± 8.18*	66.7 ± 6.83^#^	70.2 ± 4.10^#^	No
Tibial BMC (mg)	22.5 ± 2.90	23.9 ± 3.33	29.0 ± 3.55*	23.1 ± 2.43^#^	24.9 ± 1.63^#^	No

**Figure 3:**
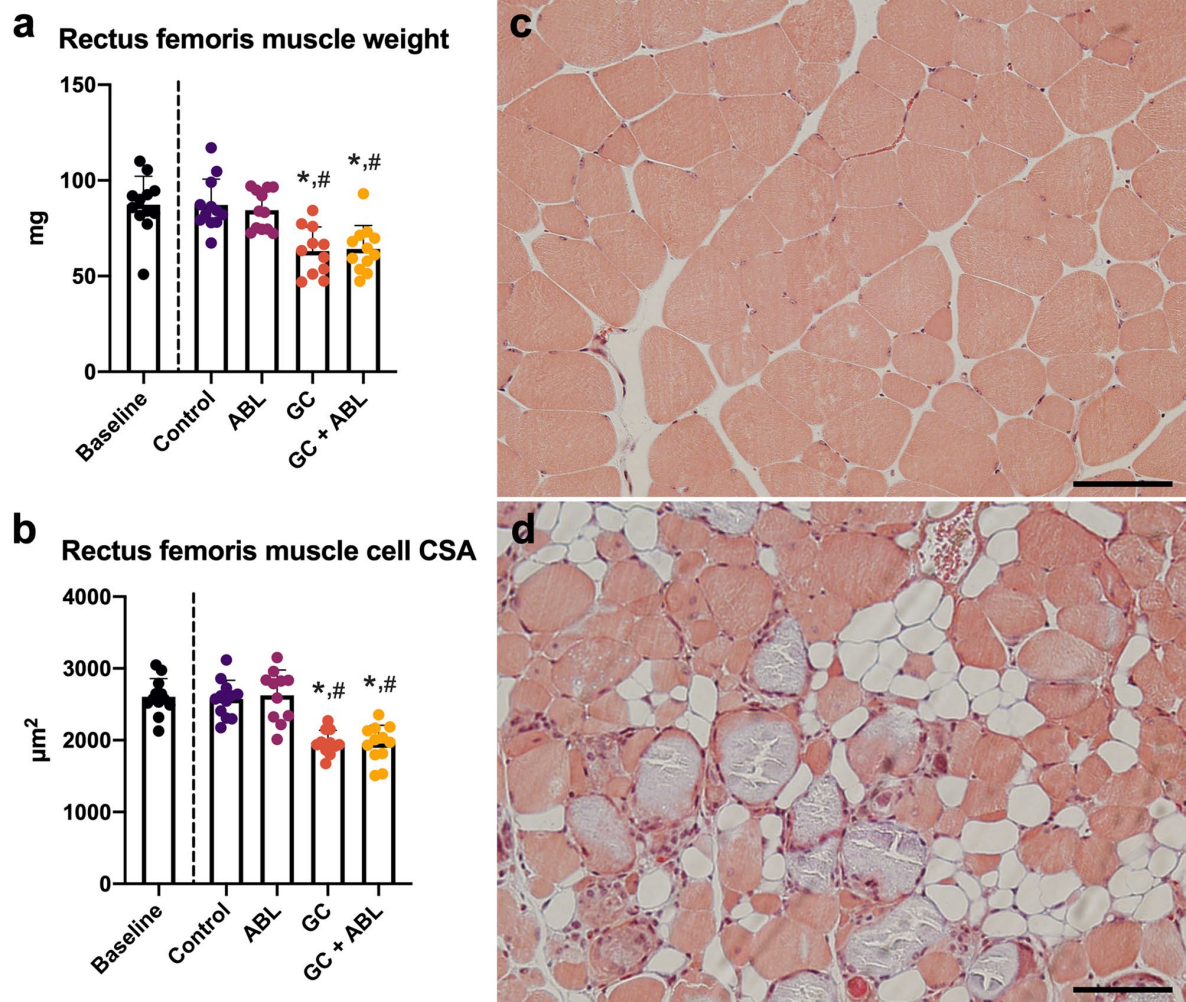
(**a**) Rectus femoris muscle mass and (**b**) rectus femoris cross-sectional area (CSA) of mice subjected to subcutaneous slow-release glucocorticoid (GC) pellets and treated with abaloparatide (ABL) for four weeks. (**c**) Representative cross-section of the rectus femoris muscle cells from a mouse in the Control group. (**d**) Representative cross-section of the rectus femoris muscle from a mouse in the GC group. Bar = 100 µm and stained with Masson trichrome. Mean ± SD. *p < 0.05 vs. Control. ^#^*p* < 0.05 vs. ABL.

As expected, treatment with ABL did not affect rectus femoris muscle weight, rectus femoris muscle CSA, or rectus femoris muscle cell CSA (Table 1, Fig. 3a,b). GC alone or in combination with ABL reduced rectus femoris muscle mass (− 28% and − 26%), rectus femoris muscle CSA (− 16% and − 25%), and muscle cell CSA (− 24% and − 24%) compared with Control, respectively. In addition, GC not only resulted in muscle cell atrophy, but also in muscle cell destruction and infiltration of adipocytes into the muscle (Fig. 3c,d).

Neither ABL nor GC affected femoral bone length (Table 1).

### Dual-energy X-ray absorptiometry

Treatment with ABL increased both femoral and tibial aBMD (+ 12% and + 13%) and BMC (+ 21% and + 21%) compared with Control, respectively (Table 1). GC did not reduce aBMD or BMC compared with Control. However, aBMD and BMC were lower at both femur and tibia in animals treated with both ABL and GC compared to animals treated with ABL only, demonstrating that GC excess blunted the osteoanabolic effect of ABL on femoral and tibial aBMD and BMC. In addition, an interaction between treatment with ABL and GC was found for femoral BMC.

### Micro-computed tomography

#### Cortical bone

At the femoral mid-diaphysis, ABL increased B.Ar (+ 21%) and T.Ar (+ 10%) compared with Control. GC alone decreased B.Ar with 5% compared with Control, as the only observable negative effect (Table 2). An interaction between treatment with ABL and GC was found for B.Ar. Treatment with GC + ABL resulted in lower B.Ar compared with ABL indicating that the effect of ABL on mid-diaphyseal B.Ar was blunted by GC. Neither GC nor ABL had any effects on mid-diaphyseal M.Ar, Ct.Po, or TMD.

**Table 2:** Morphology of the right femoral mid-diaphysis of mice subjected to subcutaneous slow-release glucocorticoid (GC) pellets and treated with abaloparatide (ABL) for four weeks.

	Baseline	Control	ABL	GC	GC + ABL	Interaction
Ct.Th (μm)	207 ± 12.7	213 ± 11.3	215 ± 29.8	207 ± 15.2	198 ± 21.8	No
B.Ar (mm^2^)	1.01 ± 0.07	1.02 ± 0.06	1.23 ± 0.14*	0.97 ± 0.08*^#^	1.04 ± 0.13^#,†^	Yes *p* = 0.031
M.Ar (mm^2^)	0.98 ± 0.10	1.09 ± 0.13	1.01 ± 0.20	1.02 ± 0.16	1.08 ± 0.12	No
T.Ar (mm^2^)	2.00 ± 0.14	2.03 ± 0.17	2.24 ± 0.23*	1.99 ± 0.21^#^	2.12 ± 0.15	No
Ct.Po (%)	3.14 ± 0.23	3.00 ± 0.25	3.56 ± 1.35	3.10 ± 0.24	3.53 ± 0.62	No
TMD (mg/cm^3^)	1178 ± 15.2	1213 ± 15.5	1200 ± 15.7	1214 ± 15.9	1213 ± 21.3	No

*Ct.Th* cortical thickness, *B.Ar* bone area, *M.Ar* marrow area, *T.Ar* tissue area, *Ct.Po* cortical porosity, and *TMD* tissue mineral density.

Mean ± SD. *p < 0.05 vs. Control. #*p* < 0.05 vs. ABL. †*p* < 0.05 vs. GC.

#### Trabecular bone

At the distal femoral metaphysis, treatment with ABL increased BV/TV (+ 140%), CD (+ 287%), and vBMD (+ 126%), whereas SMI (− 63%) and TMD (− 3%) decreased compared with Control. GC did not influence any microstructural parameters compared with Control. Animals in the GC group treated with ABL showed an increased BV/TV (+ 136%) and vBMD (+ 126%) and decreased SMI (− 80%) compared with Control. In the GC + ABL group, TMD was increased (+ 3%) compared with ABL and SMI was decreased (− 69%) compared with GC. No other differences in trabecular microstructure were observed between these groups (Table 3).

**Table 3:** Microstructural properties of femoral metaphyseal and epiphyseal bone of mice subjected to subcutaneous slow-release glucocorticoid (GC) pellets and treated with abaloparatide (ABL) for four weeks.

	Baseline	Control	ABL	GC	GC + ABL	Interaction
**Femoral metaphysis**						
BV/TV (%)	13.8 ± 3.68	6.47 ± 4.32	15.5 ± 11.5*	9.84 ± 2.77	15.3 ± 5.51*	No
Tb.Th (μm)	44.2 ± 2.84	44.5 ± 7.48	42.6 ± 6.47	41.9 ± 3.91	41.2 ± 4.07	No
Tb.Sp (μm)	262 ± 50.6	396 ± 93.5	298 ± 102	384 ± 82.8	294 ± 87.4	No
Tb.N (mm^−1^)	4.23 ± 0.80	2.76 ± 0.80	4.38 ± 2.44	3.16 ± 0.64	4.01 ± 1.17	No
CD (mm^−3^)	447 ± 200	161 ± 79.0	623 ± 551*	237 ± 78.1	532 ± 385	No
SMI	1.02 ± 0.49	1.86 ± 0.68	0.68 ± 0.95*	1.17 ± 0.41	0.36 ± 0.63*^,†^	No
vBMD (mg/cm^3^)	169 ± 43.5	81.9 ± 52.8	185 ± 129*	122 ± 35.6	185 ± 63.1*	No
TMD (mg/cm^3^)	962 ± 16.1	985 ± 16.5	951 ± 28.5*	996 ± 19.1^#^	983 ± 10.7^#^	No
**Femoral epiphysis**						
BV/TV (%)	29.9 ± 4.05	23.3 ± 6.78	30.1 ± 7.84*	26.3 ± 4.09	32.6 ± 4.24*	No
Tb.Th (μm)	61.0 ± 3.75	60.0 ± 6.73	58.7 ± 8.79	58.5 ± 4.66	59.2 ± 4.81	No
Tb.Sp (μm)	207 ± 18.1	236 ± 39.4	190 ± 21.9*	223 ± 28.6^#^	189 ± 20.9*^,†^	No
Tb.N (mm^−1^)	5.89 ± 0.66	5.17 ± 1.04	6.59 ± 1.03*	5.40 ± 0.76^#^	6.66 ± 0.85*^,†^	No
CD (mm^−3^)	314 ± 77.3	229 ± 62.6	400 ± 78.8*	243 ± 57.3^#^	356 ± 142*^,†^	No
SMI	− 0.21 ± 0.23	0.15 ± 0.52	− 0.39 ± 0.84	− 0.11 ± 0.30	− 0.66 ± 0.46*	No
vBMD (mg/cm^3^)	366 ± 47.7	293 ± 81.7	367 ± 90.8*	330 ± 49.5	399 ± 46.0*	No
TMD (mg/cm^3^)	1038 ± 15.2	1049 ± 18.1	1028 ± 23.8	1059 ± 14.5^#^	1050 ± 11.7^#^	No

*BV/TV* bone volume/tissue volume, *Tb.Th* trabecular thickness, *Tb.Sp* trabecular spacing, *Tb.N* trabecular number, *CD* connectivity density, *SMI* structure model index, *vBMD* volumetric bone mineral density, *TMD* tissue mineral density.

Mean ± SD. *p < 0.05 vs. Control. #*p* < 0.05 vs. ABL. ^†^*p* < 0.05 vs. GC.

At the distal femoral epiphysis, treatment with ABL increased BV/TV (+ 29%), Tb.N (+ 27%), CD (+ 75%), and vBMD (+ 25%) and decreased Tb.Sp (− 19%) compared with Control. GC alone had no observable negative effects on the trabecular microstructure compared to Control. Animals receiving GC and ABL had an increased BV/TV (+ 40%), Tb.N (+ 29%), CD (+ 55%), and vBMD (+ 36%) compared with Control. In addition, ABL decreased Tb.Sp (− 20%) and SMI in GC-animals compared with Control. In the GC + ABL group, TMD increased (+ 2%) compared with ABL, whereas Tb.Sp decreased (− 15%) and Tb.N and CD increased (+ 23% and + 47%) compared with GC. No other differences in trabecular microstructure were observed between these groups (Table 3).

At L4, treatment with ABL increased BV/TV (+ 45%), Tb.Th (+ 10%), CD (+ 101%), and vBMD (+ 41%) and decreased SMI. As the only observable effect compared with Control, GC increased TMD (+ 4%). In animals receiving GC, treatment with ABL increased BV/TV (+ 65%), Tb.N (+ 35%), CD (+ 63%), vBMD (+ 63%), and TMD (+ 5%) and decreased Tb.Sp (− 21%) and SMI compared with Control. In the GC + ABL group, SMI and TMD increased (+ 450% and + 5%) compared with ABL, whereas BV/TV (+ 51%), Tb.Th (+ 11%), Tb.N (+ 27%), CD (+ 64%), and vBMD (+ 46%) increased and SMI decreased compared with GC (Table 4). An interaction between treatment with ABL and GC was found for SMI.

**Table 4:** Microstructural properties of L4 of mice subjected to subcutaneous slow-release glucocorticoid (GC) pellets and treated with abaloparatide (ABL) for four weeks.

	Baseline	Control	ABL	GC	GC + ABL	Interaction
BV/TV (%)	23.5 ± 5.47	18.5 ± 5.41	26.8 ± 7.36*	20.2 ± 2.88^#^	30.5 ± 4.40*^,†^	No
Tb.Th (μm)	46.5 ± 3.86	44.0 ± 3.80	48.4 ± 5.47*	43.2 ± 2.59^#^	47.9 ± 4.03^†^	No
Tb.Sp (μm)	231 ± 33.9	274 ± 53.3	244 ± 51.8	251 ± 34.4	217 ± 3.71*	No
Tb.N (mm^−1^)	4.87 ± 0.82	4.09 ± 0.88	4.94 ± 1.12	4.35 ± 0.55	5.54 ± 0.96*^,†^	No
CD (mm^−3^)	380 ± 78.2	264 ± 85.5	531 ± 162*	263 ± 53.6^#^	431 ± 145*^,†^	No
SMI	0.09 ± 050	0.40 ± 0.46	− 0.18 ± 0.59*	0.22 ± 0.26	− 0.99 ± 0.52*^,#,†^	Yes *p* = 0.030
vBMD (mg/cm^3^)	266 ± 60.1	212 ± 61.7	298 ± 80.2*	236 ± 34.1^#^	345 ± 47.2*^,†^	No
TMD (mg/cm^3^)	946 ± 16.7	940 ± 24.9	937 ± 28.6	974 ± 15.9*^,#^	983 ± 20.0*^,#^	No

*BV/TV* bone volume/tissue volume, *Tb.Th* trabecular thickness, *Tb.Sp* trabecular spacing, *Tb.N* trabecular number, *CD* connectivity density, *SMI* structure model index, *vBMD* volumetric bone mineral density, and *TMD* tissue mineral density. Mean ± SD. *p < 0.05 vs. Control. ^#^*p* < 0.05 vs. ABL. ^†^*p* < 0.05 vs. GC.

These findings, from three different trabecular bone sites, suggest that GC excess blunted the osteoanabolic effect of ABL for only a few of the trabecular microstructural parameters.

### Mechanical testing

Treatment with ABL increased femoral mid-diaphyseal bone strength (+ 21%) compared to Control, while GC did not affect mid-diaphyseal bone strength compared to Control. In animals receiving GC alone or in combination with ABL, the mid-diaphyseal bone strength was lower (− 28% and − 26%) than in those treated with ABL alone, respectively (Table 5). An interaction between treatment with ABL and GC was found for mid-diaphyseal bone strength.

**Table 5:** Bone strength of the femoral mid-diaphysis, femoral neck, and vertebral body of L4 of mice subjected to subcutaneous slow-release glucocorticoid (GC) pellets and treated with abaloparatide (ABL) for four weeks.

	Baseline	Control	ABL	GC	GC + ABL	Interaction
Femoral mid-diaphysis (N)	18.8 ± 1.93	21.6 ± 2.32	26.2 ± 3.44*	18.9 ± 2.95^#^	19.5 ± 3.64^#^	Yes *p* = 0.038
Femoral neck (N)	19.3 ± 2.86	18.3 ± 3.74	18.3 ± 3.37	15.9 ± 3.69	16.9 ± 3.84	No
Vertebral body (N)	26.9 ± 7.93	23.7 ± 7.11	36.0 ± 8.73*	20.5 ± 5.30^#^	39.4 ± 9.73*^,†^	No

Mean ± SD. *p < 0.05 vs. Control. ^#^*p* < 0.05 vs. ABL. ^†^*p* < 0.05 vs. GC.

At the femoral neck, the bone strength did not differ between any of the groups (Table 5).

Mechanical compression testing revealed that ABL increased vertebral bone strength in both ABL (+ 52%) and GC + ABL (+ 66%) animals compared to Control. In the GC + ABL group, no difference was observed in vertebral compression strength compared with ABL. Moreover, ABL increased bone strength in GC animals (+ 92%) compared with GC animals that was not treated with ABL (Table 5).

These findings indicate that the anabolic effect of ABL on bone strength was blunted by GC at the femoral mid-diaphysis, but not at the vertebral body.

### Dynamic bone histomorphometry and histological assessment of bone cells

#### Trabecular bone at the distal femoral metaphysis

**Figure 4:**
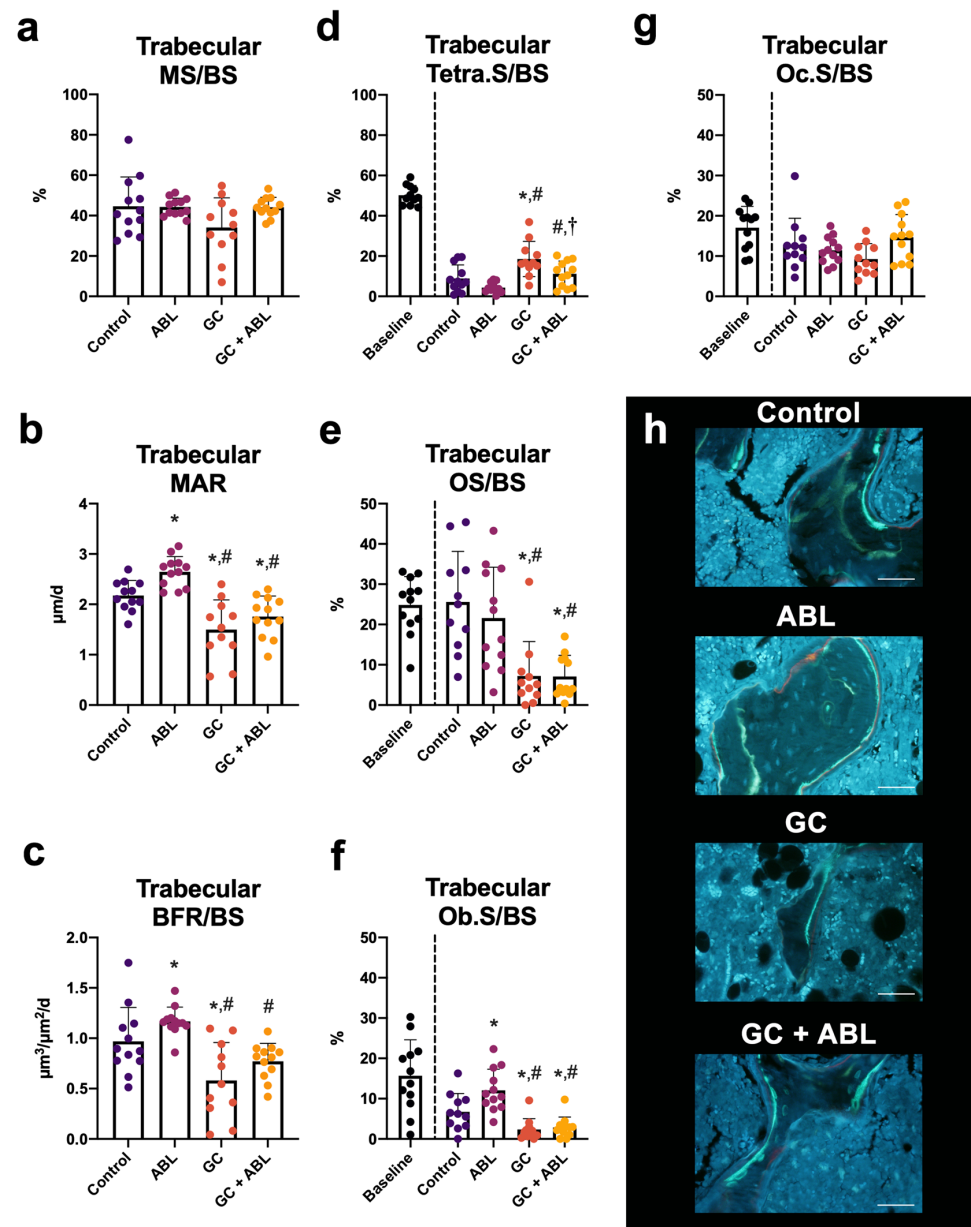
Distal femoral metaphyseal trabecular bone parameters determined by dynamic bone histomorphometry and histological assessment of osteoid, osteoblasts, and osteoclasts. (**a**) MS/BS: mineralizing surface, (**b**) MAR: mineral apposition rate, (**c**) BFR/BS: bone formation rate, (**d**) Tetra.S/BS: tetracycline-covered surfaces, (**e**) OS/BS: osteoid-covered surfaces, (**f**) Ob.S/BS: osteoblast-covered surfaces, (**g**) Oc.S/BS: osteoclast-covered surfaces, and (**h**) Representative histological sections from the four treatment groups. Bar = 50 μm. Mean ± SD. An interaction was found between treatment with ABL and GC for Ob.S/BS (*p* = 0.045) and Oc.S/BS (*p* = 0.027). *p < 0.05 vs. Control. ^#^*p* < 0.05 vs. ABL. ^†^*p* < 0.05 vs. GC.

Treatment with ABL increased MAR (+ 22%) and Ob.S/BS (+ 79%) compared with Control (Fig. 4). GC decreased MAR (− 31%), BFR/BS (− 40%), OS/BS (− 74%), and Ob.S/BS (− 66%) and increased Tetra.S/BS (+ 109%) compared with Control (Fig. 4). Animals in the GC group treated with ABL had lower MAR (− 19%), OS/BS (− 72%), and Ob.S/BS (− 57%) compared with Control (Fig. 4). Neither ABL nor GC affected Oc.S/BS. Furthermore, MAR (− 34%), BFR/BS (− 34%), OS/BS (− 76%), and Ob.S/BS (− 67%) were lower and Tetra.S/BS (+ 155%) was higher in mice treated with GC + ABL compared with mice treated with ABL only (Fig. 4). An interaction between treatment with ABL and GC was found for Ob.S/BS and Oc.S/BS. This suggests that GC blunted the effect of ABL on the resorptive parameters and hence blunted the bone turnover in trabecular bone.

The lower bone turnover in the CG and GC + ABL mice compared to the ABL mice resulted in a more mineralized bone in these animals, which is consistent with the higher TMD in these mice (Table 3).

#### Cortical bone at the femoral mid-diaphysis

**Figure 5:**
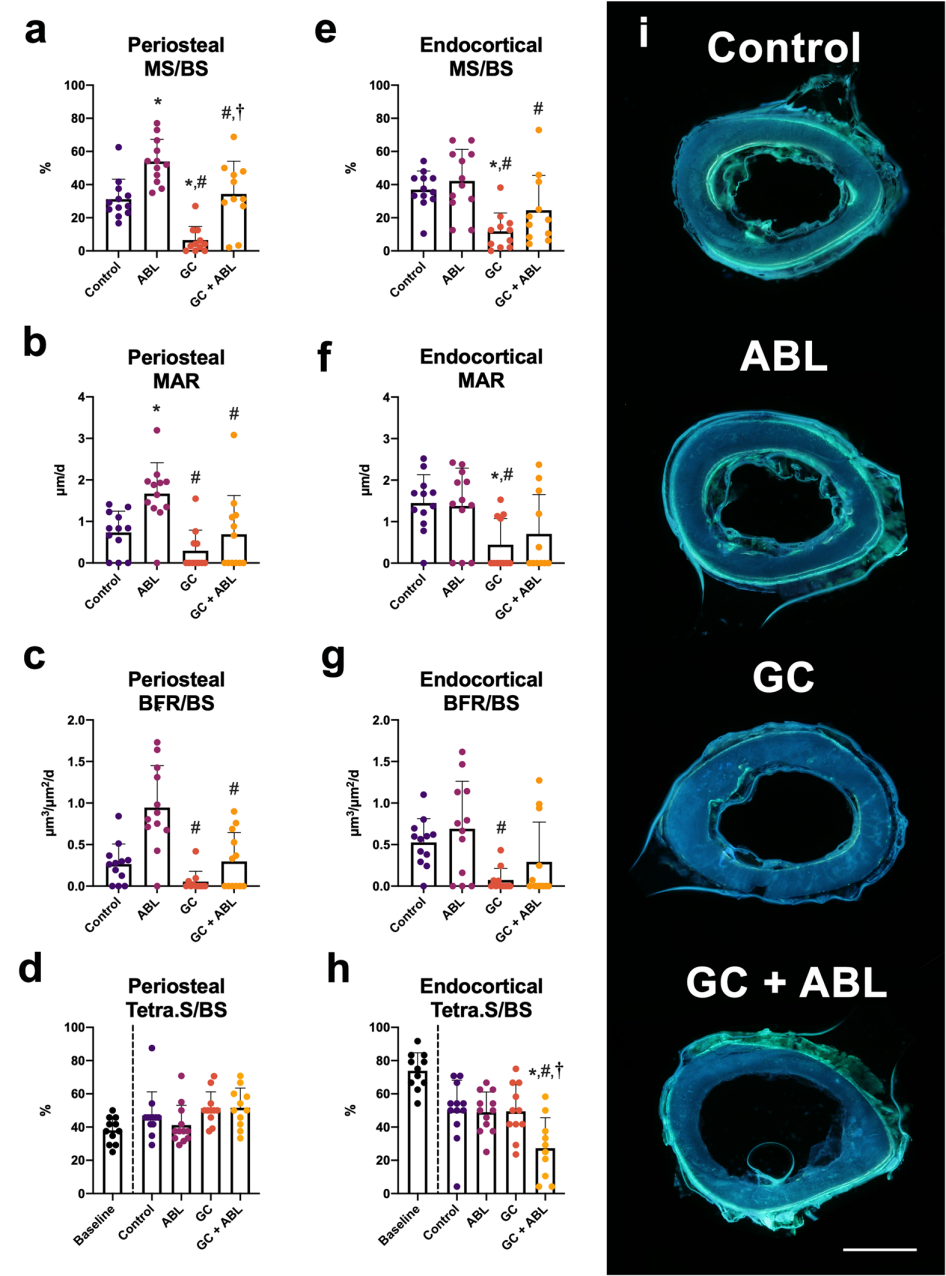
Mid-diaphyseal femoral cortical bone parameters determined by dynamic bone histomorphometry. (**a**–**d**) Periosteal envelope. (**e**–**h**) Endosteal envelope. (**i**) Representative histological sections from the four treatment groups. Bar = 0.5 mm. *MS/BS* mineralizing surface, *MAR* mineral apposition rate, *BFR/BS* bone formation rate, and *Tetra.S/BS* Tetracycline-covered surfaces. Mean ± SD. An interaction was found between treatment with ABL and GC for periosteal BFR/BS (*p* = 0.033) and endocortical Tetra.S/BS (*p* = 0.012). *p < 0.05 vs. Control. ^#^*p* < 0.05 vs. ABL. ^†^*p* < 0.05 vs. GC.

At the periosteal bone surface, ABL increased MS/BS (+ 73%), MAR (+ 128%), and BFR/BS (+ 255%) compared with Control. GC decreased periosteal MS/BS (− 79%), but did not affect either MAR or BFR/BS (Fig. 5). Animals in the GC group treated with ABL did not increase any of the formative bone parameters compared with Control. In addition, an interaction between treatment with ABL and GC was found for BFR/BS and animals in the GC + ABL group had lower MS/BS (− 36%), MAR (− 59%), and BFR/BS (− 69%) compared with animals in the ABL group suggesting that GC blunted the effect of ABL on the bone formative parameters at the periosteal femoral mid-diaphyseal bone surface.

Neither GC nor ABL had any influence on the resorptive indicator Tetra.S/BS at the periosteal surface at the femoral mid-diaphysis (Fig. 4).

At the endocortical bone surface, treatment with ABL did not influence any of the formative bone parameters assessed with dynamic bone histomorphometry in either Control or GC animals. Animals in the GC group had lower MS/BS (− 68%) and MAR (− 69%) compared with Control indicating that GC inhibited bone formation at the endocortical bone surface. An interaction between treatment with ABL and GC was found for Tetra.S/BS.

The lower Tetra.S/BS indicated that endocortical bone resorption was elevated in GC + ABL-treated mice compared with Control, ABL, and GC mice (Fig. 5).

### Adipocytes

**Figure 6:**
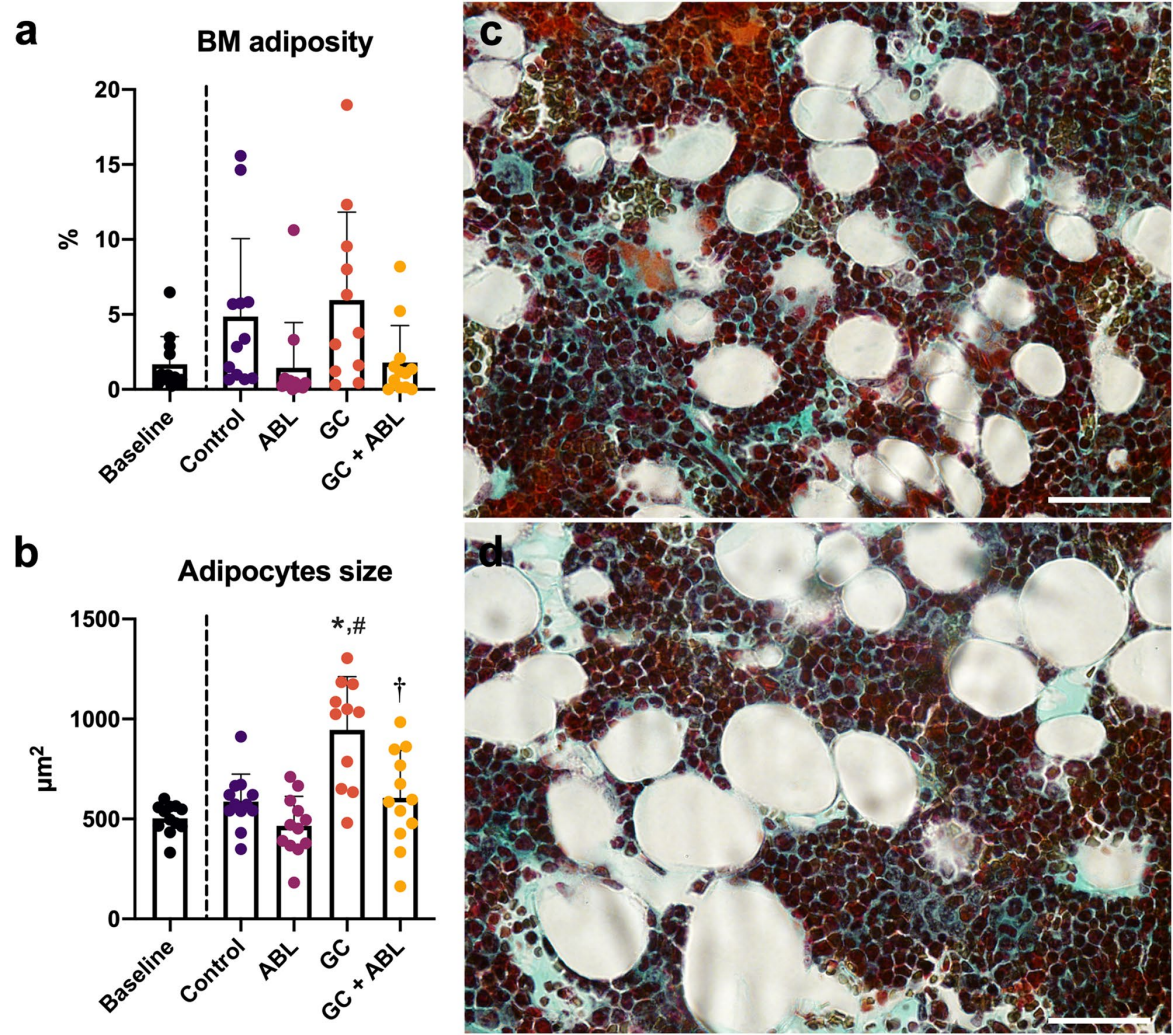
(**a**) Bone marrow (BM) adiposity, (**b**) average size of adipocytes, and (**c**,**d**) representative Masson–Goldner trichrome-stained histological sections from the distal femoral metaphysis showing adipocyte size from an animal in the Control group and GC group, respectively. Bar = 100 µm. Mean ± SD. *p < 0.05 vs. Control. ^#^*p* < 0.05 vs. ABL. ^†^*p* < 0.05 vs. GC.

A non-significant trend towards lower bone marrow adiposity in ABL treated animals compared with Control was observed. GC did not affect bone marrow adiposity, but increased adipocyte size (+ 48%) compared with Control (Fig. 6). ABL prevented the GC-induced increase in adipocyte size.

## Discussion

The purpose of the study was to investigate whether the osteoanabolic effect of ABL was affected by short-term GC excess in mice. The main finding of the study was that GC mainly blunted the osteoanabolic effect of ABL in cortical bone.

In mice not receiving GC, the potent osteoanabolic effect of ABL materialized as a substantial increase in bone mineral density, cortical and trabecular microarchitecture, and bone strength. These findings are in accordance with the results of previous studies performed in mice^36,37^. In addition, ABL increased the amount of osteoblast-covered surfaces and trended to reduce bone marrow adiposity. This suggests a possible differentiation of bone marrow mesenchymal stem cells towards osteoblasts instead of bone marrow adipocytes^38^, and is consistent with the observed lipolytic effect of PTHrP in mice^39,40^. Similar decrease in bone marrow adiposity has been reported for two other osteoanabolic agents teriparatide and romosozumab in rats^41^.

As expected, treatment with ABL did not influence muscles mass, while muscle wasting is a well-established effect of treatment with GC^42^. In accordance with that, we observed a massive reduction in muscle wet weight, muscle cross-sectional area, and muscle cell cross-sectional area in animals subjected to GC excess.

In addition to muscle wasting, GC has been shown to induce a substantial bone loss^43^. Surprisingly, we found that short-term GC excess had little or no detrimental effect on bone mineral density, trabecular microarchitecture, and bone strength.

Subcutaneously implanted pellets have been used in various mouse strains such as Swiss Webster^44–51^, CD-1^52,53^, C57BL/6^54–58^, Black Swiss^59^, and FVB/N^60^ to explore the skeletal effects of GC, but conflicting results have emerged. Several studies have reported a substantial loss of trabecular bone volume fraction or aBMD in long bones after only 21–28 days using GC doses of 2–15 mg/kg/day^44,48–50,52,54,60^, while others have reported little or no detrimental effect from implanted GC-pellets^45,59,61^. One might speculate whether the large heterogeneity in the outcomes of the different studies reflects differences in strain, GC dose, and study duration. Ersek et al. reported a GC-induced reduction in trabecular bone volume fraction, cortical thickness, and bone strength in CD-1 and C57/BL6 mice and concluded that CD1-mice (that are derived from the same mice as the RjOrl:SWISS mice used in the present study) are particularly susceptible to GC-induced bone loss compared to C57BL/6 mice. They used similarly aged mice, prednisolone pellets with a four times lower dose (2.5 mg/60 days), and a longer study duration (56 days) compared to the present study^53^. However, their findings contrast those of the present study, where GC excess had little or no detrimental effect on trabecular bone. One possible explanation for the discrepancy between the two studies is that GC-induced bone loss in RjOrl:SWISS mice requires a longer time to develop than the 28 days used in the present study or that this mouse strain is less susceptible to GC-induce bone loss. We found that GC in general had limited effect on trabecular bone, but nevertheless resulted in decreased bone resorption and formation indicators. This indicate that the microstructural changes are developing, but have not yet materialized. Furthermore, the lower trabecular bone turnover caused higher trabecular tissue mineral density in GC treated mice, signifying a more mineralized trabecular bone tissue.

In contrast to trabecular bone, GC had a noticeable effect on cortical bone. This is consistent with previous studies in mice using either GC-releasing pellets^53,61^ or subcutaneous injections with dexamethasone^62,63^, where the GC-induced bone loss was most pronounced at skeletal sites comprising cortical bone.

The present study clearly demonstrated that the osteoanabolic effect of ABL was blunted by concomitant short-term exposure to GC excess. The GC-induced blunting of ABL was more pronounced at cortical bone sites and less marked at trabecular bone sites, reflecting that GC mainly had detrimental effect on cortical bone. This might be explained by GC predominantly target cortical bone surfaces^61^, thus mainly challenging the osteoanabolic effect of ABL at the cortical bone envelopes, but not at trabecular bone surfaces.

The effect of ABL has previously been studied in botulinum toxin-immobilized rats^64^, ovariectomized rats^65^, rabbits^66^, and monkeys^26^, and intact mice^27,36,67^, but the present study is the first conducted in animals subjected to GC excess. Although no previous study has investigated the effect of ABL and GC in combination, conflicting results have emerged from studies of rodents exposed to GC and treated with teriparatide^20,62,68^. Teriparatide is structurally very similar to ABL^69^, acts through the same receptor on osteoblasts – albeit mainly in different receptor configurations^70^, and has, in general, the same osteoanabolic effect in intact mice^27,36^. One study found that GC-induced bone loss is not prevented by simultaneous treatment with teriparatide in mice^62^, while another found teriparatide effective in increasing bone mass and strength in mice subjected to GC excess by implantation of slow-release prednisolone pellets^68^. A study in rats found that GC severely blunted the anabolic effect of simultaneous treatment with teriparatide^20^.

No clinical study has investigated the osteoanabolic effect of ABL in patients with glucocorticoid-induced osteoporosis. However, one large clinical multi-center study compared the osteoanabolic effect of daily injections with teriparatide (20 μg) or ABL (80 μg) in women with post-menopausal osteoporosis^22^. They found that treatment with ABL improved femoral neck, lumbar spine, and total hip aBMD compared with treatment with placebo or teriparatide. Interestingly, the study also showed treatment with ABL entailed a lower risk of hypercalcemia than treatment with teriparatide.

At skeletal sites mainly composed of trabecular bone such as L4 and the distal femoral metaphysis and epiphysis, treatment with ABL resulted in higher BV/TV and vBMD values in both non-GC and GC exposed animals compared with Control. This suggests that GC does not blunt the osteoanabolic effect of ABL on trabecular bone density at these skeletal sites. Moreover, the increased trabecular bone density was accompanied by higher bone strength, although it was statistically significant for L4 only.

Despite GCs blunting effect on cortical bone, ABL is nevertheless a suitable candidate for counteracting GC-induced bone loss, especially when it is taken into consideration that the anabolic effect of teriparatide is similarly blunted by GC. Moreover, as treatment with ABL has shown a decreased risk of hypercalcemia, ABL provides an alternative to teriparatide and an attractive treatment option for glucocorticoid-induced osteoporosis to selected patients at risk of hypercalcemia. In addition, blunting of the osteoanabolic effect of ABL may be alleviated by increasing the dose, an option not available for teriparatide, since ABL is approved by the U.S. Food and Drug Administration to be administered at four times higher doses than teriparatide^71^.

The present study has some limitations. The assessment of bone marrow adipocytes was performed on Masson–Goldner trichrome-stained sections using cell morphology to identify adipocytes instead of using immunohistochemical staining for lipid‐associated proteins like perilipin or adipophilin. However, we have previously conducted an unpublished pilot study comparing bone marrow adiposity assessed by cell morphology on Masson–Goldner trichrome stained sections and immunohistochemical staining for perilipin and found the two methods to provide similar results. In addition, the International Bone Marrow Adiposity Society states that Masson–Goldner trichrome staining can be used to identify mature adipocytes, while immunohistochemical staining for perilipin may be used to more easily discriminate between blood vessels and adipocytes^72^.

ABL was injected five days a week due to staff conveniences, whereas in a clinical setting ABL is administrated seven days a week. It cannot be precluded that this difference may influence the anabolic effect of the ABL treatment regimen. In addition, the study was designed as a prevention study where treatment with ABL commenced when the GC pellets were implanted instead of as an intervention study where a bone loss had been allowed to develop before starting the ABL treatment.

Another limitation of the study is that GC excess was not unequivocally established by the serum prednisolone or corticosterone levels, but only *bona fide* established by subcutaneous implantation of prednisolone releasing pellets and the observable pronounced negative effect on skeletal muscle tissue. A study by Herrmann et al. used mice with the same genetic background and dosage of GC pellets as in the present study, and did not find any increase in serum corticosterone after two and three weeks of pellet implantation, suggesting serum measurements might not establish the presence of GC excess^52^.

The present study investigated short-term treatment with GC and lasted four weeks and, surprisingly, only a relatively limited detrimental effect of GC was observed for both cortical and trabecular bone. If the study had lasted longer or the dosage of GC had been higher, a more pronounced bone loss and reduction in bone strength might have occurred.

We used mice to model glucocorticoid-induced bone loss since previous studies have shown subcutaneously inserted prednisolone pellets can induce osteopenia in mice^46,57^. Mice do not show Haversian-like remodeling, but this can be achieved using rabbits, which undergo intracortical remodeling^73^. Moreover, glucocorticoid-excess in rabbits might be combined with estrogen deficiency because of the narrow therapeutic window^74,75^.

In conclusion, the osteoanabolic effect of ABL is generally blunted by short-term glucocorticoid excess in particular at cortical bone sites.
